# Overview of Policies, Guidelines, and Standards for Active Assisted Living Data Exchange: Thematic Analysis

**DOI:** 10.2196/15923

**Published:** 2020-06-22

**Authors:** Laura X Fadrique, Dia Rahman, Hélène Vaillancourt, Paul Boissonneault, Tania Donovska, Plinio P Morita

**Affiliations:** 1 School of Public Health and Health Systems University of Waterloo Waterloo, ON Canada; 2 CSA Group Toronto, ON Canada; 3 Ontario Health (East) Ottawa, ON Canada; 4 Research Institute for Aging University of Waterloo Waterloo, ON Canada; 5 Department of Systems Design Engineering University of Waterloo Waterloo, ON Canada; 6 eHealth Innovation, Techna Institute University Health Network Toronto, ON Canada; 7 Institute of Health Policy, Management, and Evaluation Dalla Lana School of Public Health University of Toronto Toronto, ON Canada

**Keywords:** ambient assisted living, active assisted living, AAL, Internet of Things, aging well, aging in place, elderly, geriatrics, standards, policies, health care, ambient intelligence, domotics, ubiquitous health, wearable

## Abstract

**Background:**

A primary concern for governments and health care systems is the rapid growth of the aging population. To provide a better quality of life for the elderly, researchers have explored the use of wearables, sensors, actuators, and mobile health technologies. The term AAL can be referred to as active assisted living or ambient assisted living, with both sometimes used interchangeably. AAL technologies describes systems designed to improve the quality of life, aid in independence, and create healthier lifestyles for those who need assistance at any stage of their lives.

**Objective:**

The aim of this study was to understand the standards and policy guidelines that companies use in the creation of AAL technologies and to highlight the gap between available technologies, standards, and policies and what should be available for use.

**Methods:**

A literature review was conducted to identify critical standards and frameworks related to AAL. Interviews with 15 different stakeholders across Canada were carried out to complement this review. The results from interviews were coded using a thematic analysis and then presented in two workshops about standards, policies, and governance to identify future steps and opportunities regarding AAL.

**Results:**

Our study showed that the base technology, standards, and policies necessary for the creation of AAL technology are not the primary problem causing disparity between existing and accessible technologies; instead nontechnical issues and integration between existing technologies present the most significant issue. A total of five themes have been identified for further analysis: (1) end user and purpose; (2) accessibility; (3) interoperability; (4) data sharing; and (5) privacy and security.

**Conclusions:**

Interoperability is currently the biggest challenge for the future of data sharing related to AAL technology. Additionally, the majority of stakeholders consider privacy and security to be the main concerns related to data sharing in the AAL scope. Further research is necessary to explore each identified gap in detail.

## Introduction

### Background

People are living longer than ever before. According to Statistics Canada’s 2016 census, seniors outnumber children aged under 15 years for the first time [1]. The aging population is growing faster than the working-age population. This adds stress on population growth, potential loss of economic productivity, and output, and a significant portion of the gross domestic product is spent on health care and pensions [2]. Aging also brings specific challenges around declining health, increase of chronic diseases, increased need for daily care and monitoring, and the financial burden of expanding health care costs. As a result, health and independence are top priorities for seniors in Canada. Active assisted living (AAL) offers technology that may address some individual and governmental concerns. In Canada, AAL technologies have been explored as a combination of smart-home, telehealth, and assistive technology (AT) [3]. In contrast, Europe has the most advanced programs for AAL standardization, and many of their projects are developed by the AAL Joint National Program [4] to support their growing aging population. Moreover, while AAL has clear applications among an aging population, similar needs exist within vulnerable populations for timely health care, monitoring, and support.

To provide better living conditions and assistance in daily routines for older adults and vulnerable populations inside and outside of their homes, researchers and innovators have explored the use of different technologies such as wearables [5], camera-based sensors [6], and mobile health technologies [7]. These new technologies and products for daily assistance allow individuals to lead a more independent life [8].

### Challenges

As mentioned by Rashidi and Mihailidis [9] in their article, the majority of older adults prefer to stay in their homes instead of transferring to a home care facility, and therefore it is essential to create and develop new technologies that support graceful aging in place. However, vulnerable populations have unique needs and limitations. Therefore, it is necessary to understand the differences and challenges in designing and developing technologies as well as collecting data for this specific user group. Standards, guidelines, and policies play an important role, ensuring better quality and safety of new technologies. In addition, standards enable knowledge sharing and act as a mechanism to make appropriately protected knowledge public and widely accessible [10].

The AAL technology landscape may seem relatively new and can sometimes be confused with the Internet of Things (IoT) as both involve data acquisition from the environment using wireless technologies [11]. IoT is the extension of the internet into physical technologies and everyday objects, which enables the creation of systems that operate over a network, collecting and exchanging data, and acting upon objects in our lives [12]. IoT devices can be used for any purpose other than health care, thus differing from AAL technologies that have the primary purpose of assisting users' quality of life. Although this new field already has many expanding technologies, AAL lacks a rigorous process of development aimed at specific users. In reviewing existing literature, some key studies on AAL technology, standards, and frameworks have been identified. The majority of the studies in this field focus on the technical specifications of AAL technologies, scenarios reviewing products, and the available tools and solutions [9,13-15]. Other studies focus on existing projects and platforms within the AAL field [2,16]. One particular study highlighted the frameworks, platforms, standards, and quality attributes of AAL technology, and so it was used as the baseline of our literature review [17].

The initial literature review and analysis demonstrated the need for researchers to understand the existing technologies and their applications. There is insufficient information on the challenges encountered in creating new AAL technologies or about ways to connect AAL technology through the use of integrated data for the benefit of the user.

### Objectives

This paper presents a review of standards, frameworks, policies, and guidelines in the creation of AAL technologies. In particular, the objective of this paper was to provide an overview of the primary standards and frameworks available as well as to highlight the gaps, challenges, and opportunities existing in the AAL area. As such, this paper did not intend to review currently existing technologies or indicate which ones to use or not. Similarly, the scope excluded any standards and frameworks targeted at medical devices even if the device is collecting personal information and is installed in a home environment to improve the quality of life. For this paper, AAL technology consists of all technology, devices, and wearables connected to the internet that enables data collection and exchange and is used for health care monitoring or to enhance the daily life of individuals.

### Active Assisted Living Concepts and Terminology

AAL technology is a subset of a broader concept called ATs, which refers to “any item, piece of equipment, or product system, whether acquired commercially, modified, or loop customized, that is used to increase, maintain, or improve [the] functional capabilities of individuals with disabilities” [18]. In other words, ATs can be any tool or device capable of assisting a person to achieve something that would not be possible without the support of that technology [8]. Wheelchairs, walkers, electronic jar openers, screen readers, hearing aids, and educational software are all examples of ATs in everyday use.

Despite being a subset of an overarching concept, AAL is also an umbrella term that describes technologies designed to improve the quality of life, aid in independence, and create healthier lifestyles for those who need assistance at any stage of their lives. AAL involves concepts, products, and services that combine new technologies and environment to improve the quality of life at all ages [19]. AAL uses information and communication technologies combined with the physical and social setting to provide easy-to-use devices either at home or to support lifestyles outside of the home environment [4]. According to the International Electrotechnical Commission (IEC) Systems Committee on AAL (SyC AAL), this technology supports systems for the elderly in industrialized countries by helping them live healthier lives [20]. The term AAL can be referred to as active assisted living or ambient assisted living as both terms are used interchangeably throughout the literature. This research report will follow the terminology defined by the SyC AAL committee that defines AAL as active assisted living technology [20]. The active assisted living terminology was chosen because it goes beyond the ambient and can be used outside the residence such as a smart walker.

Technology could prove highly beneficial in providing a higher quality of life for an aging society or for anyone who needs additional help to perform their daily tasks and activities inside or outside their homes. To address this matter, the International Medical Informatics Association of the United States approved the creation of a new workgroup on smart homes and AAL in November of 2006 [21].

The AAL environment can integrate multisensors inside or outside of the home to gather data and monitor individuals in their homes [22]. The integration of sensors, embedded in homes, is also known as ambient intelligence (AmI) [23]. AmI applications should be transparent to users while meeting security and privacy requirements [24]. Many current devices, sensors, and health/wellness trackers are capable of collecting and sending information to a caregiver or physician for remote patient monitoring. However, the creation of new solutions for the aging population requires special attention, should not rely on the user’s effort, and needs to consider the cognitive, perceptual, or physical limitations of users [9].

AAL technology devices can be either simple, such as presence sensors, or complex such as a smart wheelchair controlled via eye movements. AAL technologies are not subject to the same rigorous standards and evaluation protocols required for medical devices. Standards are widely used by companies in the planning, development, and production of these products and technologies. Without standards, possible interactions between products could be inconsistent, processes would not be defined and secure, and there would be security and safety-related risks [25]. For the Institute of Electrical and Electronics Engineers (IEEE), standards are “published documents that establish specifications and procedures designed to maximize the reliability of the materials, products, methods, and/or services people use every day” [26]. This project focused on identifying existing standards relevant for AAL technology and explored the existing gaps in terms of standards for supporting the development of AAL technologies, and to do that we interacted with different stakeholders in this space. In addition to standards, it is necessary to explore protocols used within the standards. In this case, protocols are a set of rules or procedures for the way information will be structured and transmitted for electronic devices to send and receive data [27].

## Methods

### Study Design

This project was planned in three phases. The first phase focused on conducting a literature review to understand what currently exists regarding standards and guidelines for AAL technologies. The second phase interviewed key industry stakeholders to develop a better understanding of the use of standards, the use of data-sharing practices, and the challenges of AAL technology to identify the existing gaps in the development of AAL technologies. The third phase aimed to validate the results of the literature review and interview phases through conducting a workshop. The interview method was chosen because it allows more feedback points to be collected from a single individual [28]. As for the workshops, participants promoted group discussions and elaborated on each other's responses, influencing the direction of the workshop [29]. However, the workshops by themselves have disadvantages around the existence of dominant people that dominate the discussion vs interviews that allow users to voice their opinion with regard to that dominance [30].

It is essential to mention that standards or guidelines related to medical devices and their safety were excluded from the scope of the project as there are existing regulations and standards in place to support the development of these devices.

### Literature Review

To meet the objectives of this project, a narrative literature review was performed to understand the existing material regarding technical standards, frameworks, and platforms related to AAL technology. Databases used included Scopus, IEC, IEEE, and PubMed as the primary sources for academic references and standards references. The IEC and IEEE databases were selected from the collection of publications related to engineering and computing standards and guidelines. Scopus and PubMed were used to review publications regarding science, technology, health care systems, and medicine. The academic literature led to an in-depth evaluation of gray literature and websites from AAL governmental programs around the world. Furthermore, results from the academic literature leverage the creation of the questions for the semistructured interview used in the next phase of the project. The technology-oriented standards covered in this research report were driven by the research conducted by Memon et al [17] in the article titled “Ambient Assisted Living Healthcare Frameworks, Platforms, Standards, and Quality Attributes;” a website from Postscapes [31] called “IoT Standards and Protocols;” and Salman’s [32] paper titled “Networking Protocols and Standards for Internet of Things.”

### Interviews

In the second phase of this research project, we used a semistructured interview (see Multimedia Appendix 1) with 12 to 17 questions. The semistructured method uses a list of predetermined questions that guide the interview and may or may not be used according to the course of the conversation and previous answers. This method brings out how the interviewee interprets the topics and problems presented [28]. Over 50 stakeholders in AAL technology, from Canada, were invited to participate in interviews. Stakeholders were selected among four distinct categories: (1) health care providers such as physicians, nurses, and social workers; (2) academics and researchers who represent a large percentage of the stakeholders in the field; (3) industry representatives from well-established corporations to small start-ups working to innovate and find better ways to help people; and (4) health care administrators responsible for decision making in research or acquisition of new technologies. The stakeholder list was formed along with a project advisory panel that identified and suggested the names of experts from across Canada with some interest or involvement with AAL. The stakeholders were then divided into the suggested categories.

A round of invitations were sent to all stakeholders, along with an information letter and a description of the project objectives. A total of 15 invited participants agreed to be interviewed. A date and time were scheduled for each participant. The interviews were conducted over the phone by 2 researchers. Each phone call lasted approximately 60 min and was recorded. After a brief introduction of the project, the respondents were presented with approximately 17 questions (the questions could vary according to previous answers) on four distinct areas: “What is AAL?;” ”Standards;” “Data Sharing;” and ”Main Challenge.”

The interview results were coded using a thematic analysis because it is best suited to identifying topics within verbal or written interviews using semistructured interviews [33,34]. Furthermore, the data were analyzed using the 6-phase approach to a thematic analysis proposed by Braun and Clarke [35]. We identified saturation on our themes, instead of saturation on the data.

### Workshops

The literature review and interview phases generated a list of standards, platforms, and frameworks, as well as a list of topics. These results were then presented at a workshop organized to fulfill the predefined purpose of validating the results and leverage new insights into and suggestions on the topics presented. A second round of emails were sent to more than 70 stakeholders from Canada, inviting them to participate in a face-to-face workshop with the possibility of online participation, and a total of 11 participants attended the workshop conducted in April 2018. The workshop was created in an unstructured manner where the project researchers acted as facilitators guiding the session. Participants were given the opportunity to present their ideas related to the topics presented and to challenge other participants’ ideas. The expected outcome was to identify the collective understanding of the topics presented and thus build a common meaning and validate the results.

Future steps and opportunities related to AAL technology were identified as a result of conducting the workshop.

## Results

### Standards

**Figure 1:**
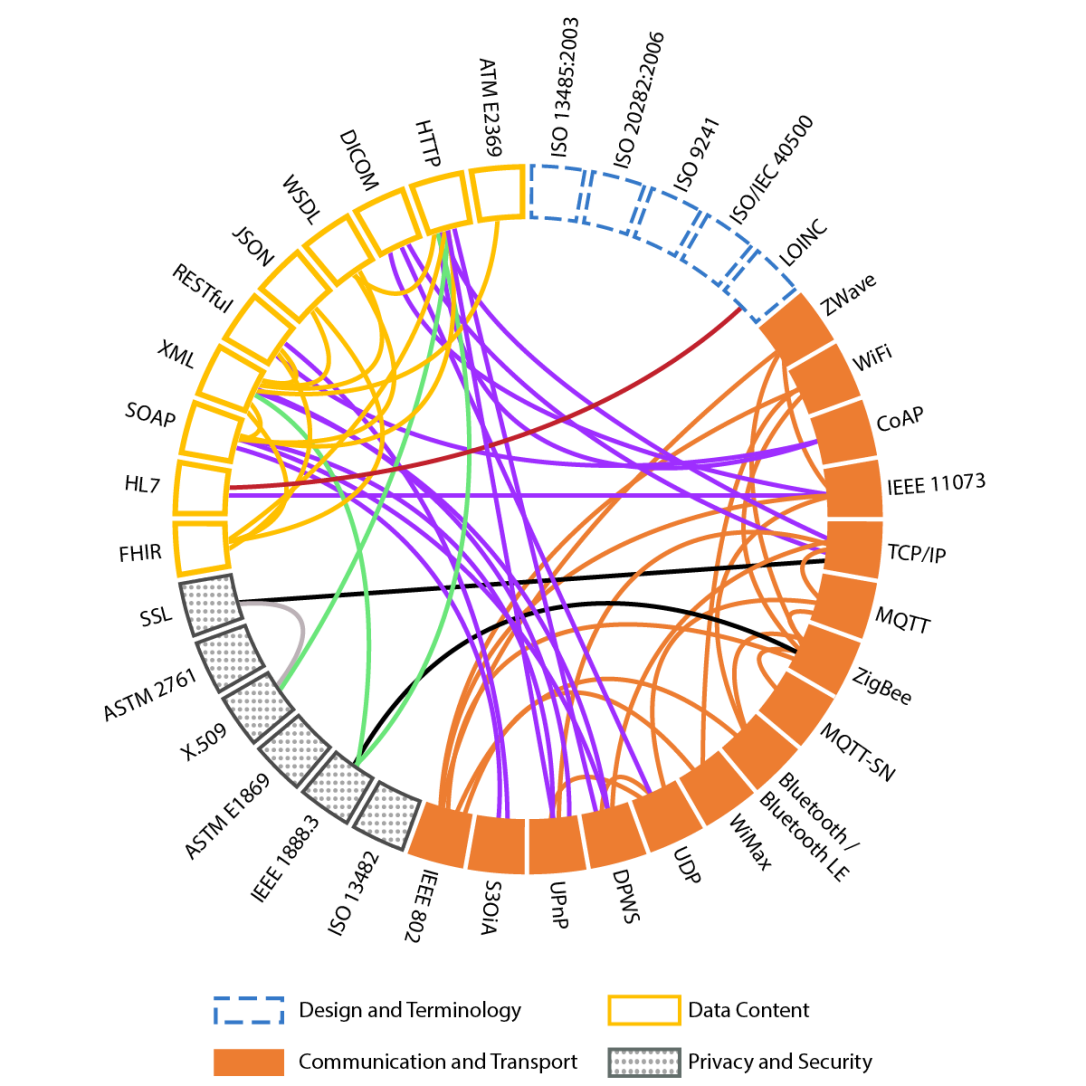
Relationship between standards and protocols.

Considering a variety of possible information technology standards, the literature review showed that standards related to essential technologies, hardware, devices, application programming interfaces, and middleware are well-covered by the existing standards of leading institutes such as IEEE, International Organization for Standardization (ISO), and IEC. On the basis of this information, the identified standards relevant to AAL technology were grouped into the four following categories: (1) design and terminology; (2) communication and transport; (3) privacy and security; and (4) data content. For this study, the design and terminology group was responsible for representing concepts using correct terminology and processes related to design, modeling, and planning. Any standards responsible for ensuring the information were transmitted reliably, and independent of the message sent, they were classified as communication and transport. Privacy and security standards are responsible for setting administrative, physical, and technical actions to protect the confidentiality, availability, and integrity of the information. The data content group contains standards responsible for the transferred information and data format that usually uses existing communication protocols. The frequencies of the different groups are shown in Table 1, where the first column displays the standard group and the second column lists the number of standards or protocols identified for a given group as well as the percentage relative to the total. Figure 1 represents the primary standards and protocols investigated in this report and the existing association between them. The figure is a radial chart, sectioned in four categories. The top-left area (solid yellow line with no fill) shows the standards responsible for data content; the top-right area (dotted blue line with no fill) shows patterns used for design and terminology; the bottom-left area (solid grey line with pattern fill) shows the security and privacy standards; the bottom-right area (solid orange fill) shows patterns related to communication and data transport. The link between the standards can represent a dependency—in this case, one standard does not exist without the other—or the indication that one standard is based on another one.

**Table 1:** Number of standards for each standard category identified (N=43).

Standard group	Total, n (%)
Design and terminology	5 (12)
Communication and transport	15 (35)
Privacy and security	13 (30)
Data content	10 (23)

### Framework and Platform

Beyond the standards and protocols, several frameworks and platforms were identified as relevant for the AAL context. These frameworks bring together multiple standards and guidelines, thus enabling products compatible with the platform or framework to integrate with other products of the same family with ease. Some frameworks or platforms were created for health system purposes or specifically for AAL technology, including the following:

1. *Continua framework*: Continua is a framework created by the Personal Connected Health Alliance based on open standards. The framework is known as a guideline for the safe, secure, and reliable exchange of data to and from personal health devices [36].
2. Persona platform: Persona platform is an integrated project financed by the European Commission on AAL for an Aging Society to develop sustainable and affordable solutions for senior citizens to live independently [37].
3. UniversAAL: UniversAAL is an open-source middleware platform for AAL that enables the rapid development of innovative IoT solutions [38].
4. ZigBee Healthcare: ZigBee Healthcare (proprietary) is an AT that is designed to be simple and easy for users to maintain their independence and mobility. This type of network can connect to sensors and controllers without being restricted by distance and range [39].
5. Microsoft HealthVault: Microsoft HealthVault is a proprietary platform built by Microsoft to support the collection, storage, use, and sharing of health information for users, family members, and care providers. It allows all health information to be accessed from a single place.
6. IEC/SyC AAL: The IEC SyC AAL has developed an international roadmap for standards for AAL systems and services to ensure safety, security, privacy, and interoperability [20].
7. Apple HealthKit: Apple HealthKit is a proprietary platform available for iPhone users to collect and aggregate health data via wearables and apps that are installed or synchronized with the users’ iPhones or Apple Watch. HealthKit provides a standardized framework for the storage and sharing of health information, allowing users to control their data access and integration [40].

### Interviews and Workshops

Interviews have highlighted that approximately 40% of the interviewees were well versed with the term AAL, while 27% had heard of the term but could not explain the meaning of the abbreviation and 33% did not know the terminology. After a brief explanation of the AAL concept, 60% of the interviewees already knew the idea even though they were not familiar with the specific terminology. The interview participants also confirmed that the terminology is considered a problem, with the first issue being the acronym AAL that can mean either *ambient assisted living* or *active assisted living* depending on the particular context or group. Another concern is regarding the existing stigma with the term *assisted* because it implies the need for assistance and support, which several technology users do not desire. These findings were confirmed by the workshop participants as well.

Questions related to standards showed that all the interviewees agree on the use and creation of specific standards and guidelines of AAL technology. Participants in academia pointed out that even though standards are not fully applicable in the research area, they are of paramount importance in the development of new technologies. In addition, standards-related responses showed concerns with end users, user safety, product accessibility, and the purpose of using the technology.

When questioned about data sharing, privacy and security were identified as the major challenge. The participants also expressed concern about proper interoperability between products to ensure the correct exchange of information. However, all participants agreed that they would share their data for research and to improve public health if adequate safety policies were implemented and data anonymity is practiced. Another point raised was related to data accessibility, especially in the context of the elderly and vulnerable populations.

Regarding the challenges of creating new AAL technologies, most participants understood that technology challenges or lack of technology is not the problem in the creation process. If the technology does not exist yet, it will probably be created. The problem lies in ensuring security, privacy, proper data sharing, product interoperability, and the correct use of technology. It is essential to understand the purpose of the product being developed to be able to select appropriate technology to ensure the greatest benefit to the end user.

### The Five Challenges

**Figure 2:**
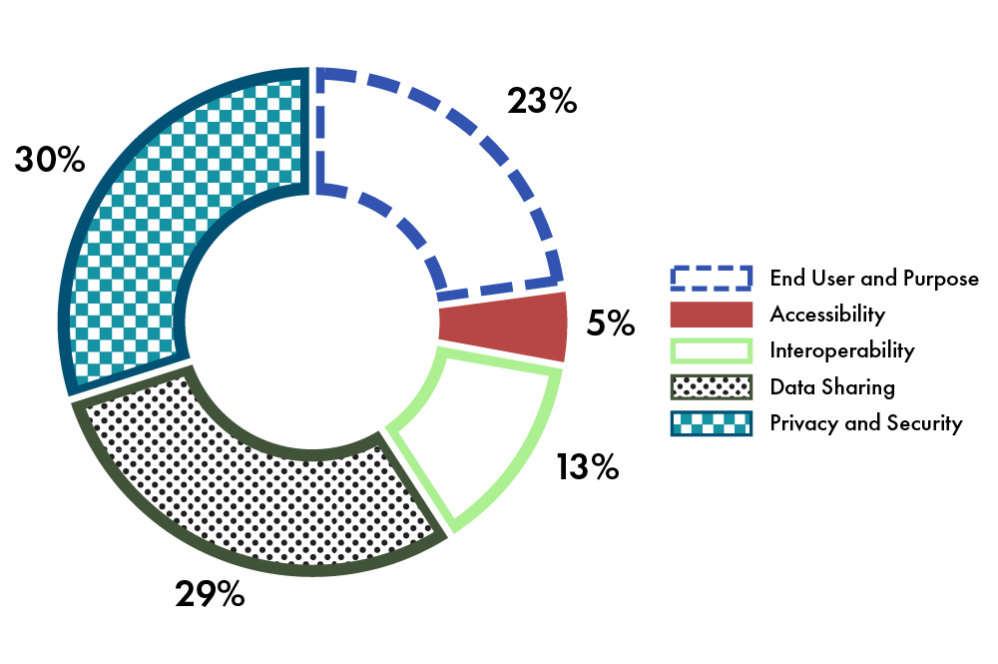
Frequency of gaps in the interviews and workshop.

After the round of interviews, the notes were analyzed together with a revision of the standards and framework, and five significant gaps were identified: (1) end user and purpose, (2) accessibility, (3) interoperability, (4) data sharing, and (5) privacy and security. Table 2 shows the number of mentions for each gap by the type of stakeholder. Home care administrators are primarily concerned with the benefits for the end user and if the proposed technology does what it is intended to do, while the industry and health care providers place more value on privacy and security. Furthermore, academics mention data sharing as the biggest challenge. Figure 2 shows the frequency of each gap mentioned during the interviews and workshops. Privacy and security is the primary issue, with 30% of mentions, followed by data sharing with 29%, and end user and purpose with 23%. Accessibility is the least mentioned gap with 5%, and the second-lowest gap is interoperability with 13% of mentions. Although interoperability is at the end of the list, it is identified in the literature to be the most significant technical challenge within AAL technologies.

**Table 2:** Number of times a gap was mentioned by a group of stakeholders—interview result.

Gap group	Accessibility	Interoperability	End user and purpose	Data sharing	Privacy and security	Group total, N
Health care providers, n (%)	1 (14)	0 (0)	1 (14)	2 (29)	3 (43)	7
Academic, n (%)	1 (4)	3 (13)	4 (17)	10 (43)	5 (22)	23
Industry, n (%)	1 (3)	4 (13)	6 (20)	7 (23)	12 (40)	30
Home care admins, n (%)	1 (4)	4 (17)	8 (35)	5 (22)	5 (22)	23
Gap total, n	4	11	19	24	25	83

#### End User and Purpose

AAL technologies are meant to assist users and keep them and their data safe. There is an overall perception that there is not enough consideration for the end user during product development. The end user should participate in all the phases of development, helping in the planning, designing, and testing of new technologies to ensure that their specific impairments, diseases, and disabilities are being accurately addressed. The technology should be adaptive to the context and satisfy the users’ needs. Another concern raised by the participants is that a significant portion of the technology available in the market was not developed to solve a clinical problem. Instead, it was adapted from the original purpose (eg, home security) to solve an alternate problem.

#### Accessibility

The access to and use of technology, regardless of user ability, is an essential aspect of AAL technology. Accessibility is one of the themes identified by interview and workshop participants as differentiating average users from AAL users. The word accessibility has emerged as one of the top 5 significant gaps related to standards and guidelines within the scope of AAL technology. Creating products that are accessible to all is where the effort must be concentrated to ensure maximum benefits from technology. Lack of accessibility can lead to a decrease in device acceptability due to a deficiency of support for AAL users' needs.

#### Interoperability

The lack of interoperability between AAL technologies was one of the most predominant technical challenges in the reviewed literature and among the participants interviewed. Owing to the lack of regulation, the manufacturer enables integration with other devices that use the same protocol when choosing a solution (eg, the ZigBee protocol), disabling integration for devices that have opted for other alternatives. Many of the standards, protocols, and framework presented in this paper have the goal of solving this problem.

#### Data Sharing

Standardizing the data-sharing process between devices is one of the major challenges in the field of AAL technologies. Most devices cannot communicate with each other due to a lack of proper interoperability. Even when they do use the same technology, data exchange is not always feasible. As such, the challenges related to data sharing between AAL devices are related to the availability, reliability, integrity, validity, and accuracy of the collected data. In particular, how to ensure that data collected on one device are transmitted to another device without loss of information and quality. The interviews and workshops reveal that it is necessary to work with the terminology of the data so that the information is significant and creates unified terminology models across all manufacturers. In doing so, it is possible to transform data and present the results to the end user in a clear and understandable way without the need for technical or specialized knowledge for data interpretation.

#### Privacy and Security

Security concerns range from technical issues—whether devices are protected against viruses or hackers—to ensuring that devices are designed and developed with security in mind by choosing the best algorithms and encryption available. Devices that serve more than one purpose run a higher risk of having security and privacy requirements that are not correctly designed. Ensuring that the data collected are properly anonymized and aggregated where appropriate, so as not to pose a potential risk to the end user, is the most significant privacy concern. Most participants report the need for a transparent process that clarifies how data flow across the internet or devices, which data are being collected, and how the data are used. There should be a clear explanation of when and how data are shared and who has access to the information. Trust will only be achieved with proper end-user education, transparency, and accessible presentation of end-user policy contracts.

## Discussion

### Overview

This study used literature reviews, interviews, and workshops to identify existing patterns, structures, and guidelines for the development of AAL technology, as well as to identify existing gaps in the area.

Our research has shown that the Canadian aging population could benefit from the innovation of AAL technology in the coming years. AAL technology can provide solutions to increase the security and independence of the population, as well as improve the quality of life, allowing seniors to age in their homes. Therefore, it is crucial to continue investing in projects and solutions to improve the development of AAL technologies.

Interestingly, the results showed that most of the challenges within the scope of this problem are not related to the availability of technology but to the way technology is applied to solve current problems.

### Literature Review

Our findings from the literature review showed that most technical standards such as ZigBee, Z-Wave, Bluetooth, ISO/IEEE 11073, and others listed in the standards section of this report are already available or currently in development and applicable in the development of AAL technologies. Different organizations are working on arranging technical standards in specific frameworks. An example is the IEEE 2413—IoT architecture, which is a unified approach to the development of IoT systems and the ISO/IEC JTC 1/SC 41—IoT and related technologies that include sensor networks and wearable technologies.

The list of privacy-related standards for IoT technologies related to AAL technology was limited when the literature review was initiated in September 2017. Only 1 year later, more than 10 ISO privacy standards relevant to AAL were found under development. This evolution in standards addresses one of the concerns identified during the study—that technology evolves very quickly, and innovation is an ongoing process. Guidelines on which technologies and structures to use can very quickly become obsolete or subject to unnecessary bureaucratic intervention.

Although the literature review has resulted in a list of existing technical standards, or under development, for use in the scope of AAL technologies, nontechnological issues have the most gaps in terms of standardization. Issues related to the human interface, processes and methods, vocabulary and social and cultural norms require special attention. Currently, there is no common terminology; hence, finding common terminology is one of the most critical areas to be consolidated in the AAL domain. Similarly, it is necessary to identify the requirements for all possible use cases, the need for specific human processes and interfaces, and create appropriate standards for each scenario. Furthermore, user engagement is widely accepted as an essential concept in the development of new systems and technologies and should be extended to the development of AAL technologies.

The design process of new AAL technologies requires special attention due to the cognitive, perceptual, or physical limitations of the target users. Such technology should not depend solely on the user’s effort and input but rather create automated solutions with minimal interaction. Yet, it is essential to take privacy considerations and concerns seriously and discuss these issues with users. Incorporating the end user in the early design stages is critical to increasing the acceptance of technology, and it is an essential part of avoiding unexpected user experience conflicts.

In this field, it is crucial to understand that the concept of end users is not limited to patients, older adults, and people with disabilities or specific health problems. Users also include therapists, health care providers, physicians, and family members who support the daily routines of vulnerable populations through the use of technology.

### Interviews and Workshops

When talking to interviewees about standards and challenges in the AAL environment, the need to go beyond the technical aspect became clearer. Some challenges include creating goal-oriented and user-friendly solutions, understanding the user’s needs, and choosing the right technologies to meet those requirements. AAL technology is directly related to the intended use of the device. Each specific use case may require different details, standards, design, security, access, and data sharing. The same device that is usually considered consumer goods may, in another scenario, serve as an assistive device to an elderly or a vulnerable individual if it can improve their quality of life. Therefore, the definition of AAL technology becomes a challenge, as many consumer technologies could have AAL applications. For example, Google Home, a smart-home system, is not immediately identified as AAL technology, but it can also be used by individuals with special needs to control light switches because they cannot reach them. In this case, there is the adaptation of existing technology used to address a particular need. Adapting technology that is not designed for a specific use could put the end user at risk and compromise safety and trust.

In addition to the consideration for the end user, the purpose of the technology, accessibility, and privacy, interoperability remains to be one of the main challenges of AAL technology. The integration of products from different manufacturers through common standards will not happen without significant effort from governments and standardization agencies. Furthermore, the use of data collected at a population level for public health analysis and improvement of overall health has the potential to provide value to the data currently being collected by multiple devices. This analysis has the potential to aggregate individualized data and extracts meaning. Innovators should focus on making raw data more understandable and relevant to users and clinicians by providing context for the collected data.

It is necessary to address the concern of whether the users, health care providers, family members, and technology itself are collecting and storing data correctly, securely, and with sufficient data quality for clinical use. The creation of guidelines to ensure data reliability, trust of the data source, and trust in the process of aggregation and analysis will be critical to enable the integration of AAL technology data into clinical practice. Another critical issue is individual literacy. The focus needs to be on educating the public about AAL technology and getting them to be aware of the benefits of existing solutions in the market. Educating target users, influencers, health care providers, and the local community to guide families to better understand AAL technology and its uses can be a viable solution.

In summary, ethics, user friendliness, user acceptance, economic benefit, legal challenges, and data privacy have to be considered to provide sustainable and well-accepted AAL solutions in Canada. Additionally, more interorganizational collaborations and user-focused studies are necessary to explore the benefits of AAL technology in Canada.

### Conclusions

Although adopting a set of standards may not address all of the gaps identified in this paper, they are essential tools that can be combined with regulations, policies, and programs to promote change. Various opportunities have been identified in this report through an extensive literature review and stakeholder consultations through interviews and workshops. User friendliness, user acceptance, and data privacy have to be considered to provide sustainable and accepted AAL solutions in Canada. Interorganizational collaborations and user-focused studies are necessary to explore the benefits of AAL technology for Canadian citizens and to ensure that this technology makes a significant positive impact on our health care system. Further in-depth research is needed to explore the existing gaps in AAL technologies.

## Appendix

Multimedia Appendix 1 Interview questions.

## Abbreviations

AAL: active assisted living
AmI: ambient intelligence
AT: assistive technology
IEC: International Electrotechnical Commission
IEEE: Institute of Electrical and Electronics Engineers
IoT: Internet of Things
ISO: International Organization for Standardization
SyC AAL: Systems Committee on Active Assisted Living
