# Supplementary material for: Overview of Policies, Guidelines, and Standards for Active Assisted Living Data Exchange: Thematic Analysis
Source: JMIR Mhealth Uhealth. 2020 Jun 22;8(6):e15923. doi: 10.2196/15923 (PMC7338926; doi:10.2196/15923)
Supplement: Multimedia Appendix 1 [file mhealth_v8i6e15923_app1.docx]

# Appendix A – Interview Questions

1. Are you familiar with the term Ambient Assisted Living (AAL) term?
2. How would you define AAL?
3. What is your involvement with AAL?
4. Are you involved in the decision-making of AAL products? Describe your involvement.
5. What do you think of the use of standards and guidelines in creating new products?
6. Did you adopt any known standard in your development? List all the standards used.
7. How did you choose the standards to use?
8. Which standards were missing that could have helped your product development?
9. Did you consider similar products as baseline for your product?
10. Do you agree to the creation of specific standards for AAL technology? What standards specifically?
11. What are your thoughts about data-sharing?
12. Did you be willing to share the data collected in your product to improve health conditions of the population?
13. Which was the most challenging area to work with in the creation of an AAL product?
    - Infrastructure
    - Hardware
    - Software
    - Security
    - Policies
    - Explain why?
14. What were the security challenges you faced during planning and development?
15. Did you have difficulties to find and implement best practices?
16. Was security a problem (hardware, data, connection)? Describe why.
17. How you think an AAL standard should be?
